# Evaluation of Depression and Anxiety in Coronary Artery Bypass Surgery Patients: A Prospective Clinical Study

**DOI:** 10.21470/1678-9741-2018-0426

**Published:** 2019

**Authors:** Melike Elif Teker Açıkel

**Affiliations:** 1 Department of Cardiovascular Surgery, S.B.Ü. Haseki Education and Research Hospital, İstanbul, Turkey.

**Keywords:** Depressive Disorder, Anxiety Disorders, Coronary Artery Bypass, Coronary Artery Diseases, Quality of Life

## Abstract

**Objective:**

The aim of this clinical study is to determine the depression and anxiety levels in coronary artery bypass graft (CABG) surgery patients in the pre and postoperative periods.

**Methods:**

This clinical prospective study was done with 65 patients. Beck’s Depression Inventory (BDI) and Beck’s Anxiety Inventory (BAI) tests were performed in patients who had a diagnosis of coronary artery disease and were awaiting CABG surgery. These patients presented characteristic symptoms of anxiety and depression and BDI and BAI tests are important to assess these symptoms.

**Results:**

We found out that depression and anxiety levels were higher in the postoperative than in the preoperative period (*P*<0.001). Both anxiety and depression levels were increased significantly following CABG operation when compared with preoperative levels in all patients. Statistical correlation of depression and anxiety in different ages, genders, and professions were evaluated too, but we did not found a correlation between them (*P*>0.05).

**Conclusion:**

We suggest that good management of the psychological condition of cardiac surgery candidates, as well as post-bypass patients, will improve quality of life and cardiovascular outcomes in these patients.

**Table:** 

Abbreviations, acronyms & symbols	
ANOVA	= Analysis of variance
BAI	= Beck’s Anxiety Inventory
BDI	= Beck’s Depression Inventory
CABG	= Coronary artery bypass graft
CAD	= Coronary artery disease
SD	= Standard deviation
SPSS	= Statistical Package for the Social Sciences

## INTRODUCTION

Coronary artery bypass graft (CABG) surgery is still the best treatment for multivessel and left main disease when considered the survival, improved ventricular function, freedom from recurrent angina, and reintervention rates^[1,2]^. However, CABG operation negatively affects the psychological condition of the patients, because of their thinking about pain and the risk of death. Furthermore, they are separated from their family, their friends, and their professional life during the preoperative and postoperative periods. The ınability to adapt to this situation results in increased anxiety and depression^[3]^. The aim of the present study is to determine the depression and anxiety levels in CABG surgery patients in the pre and postoperative periods. We also evaluate the symptoms of depression and anxiety in different age, sex, and professional groups.

## METHODS

This clinical prospective study was done with 65 patients. These patients had undergone CABG for one year and did not use psychiatric medication.

Beck’s Depression Inventory (BDI) and Beck’s Anxiety Inventory (BAI) tests were performed in patients with diagnosis of coronary artery disease (CAD) and were awaiting CABG surgery. Fifty of these patients were males, and fifteen were females. Their average age was 61.0±11.7 years. Patients’ demographic data are on Table 1. Fifteen of the patients were housewifes, 19 were retired, 17 were self-employed, and 14 had another profession. Patients were taken to a quiet room with the doctor. The doctors asked questions from the BDI and BAI tests to the patient. The patients answered both the BDI and BAI tests. The answers were marked by the doctors on the tests. The patients were admitted to the cardiovascular surgery clinic two days before the operation. The tests were done on the preoperative 1^st^ day and postoperative 3^rd^ day with in-hospital patients and on the postoperative 7^th^ and 30^th^ days with out-hospital patients. The exclusion criterion was the presence of hemodynamic instability. No patient reported use of psychotropic drugs. No patient had any chronic psychological illness. The medical history of the patients was obtained and ıt was decided whether or not to be included in the study. The study protocol was approved by the institutional Ethics Committee of Haliç University (2018/09-82-10). The study was conducted in accordance with the principles of the Declaration of Helsinki.

**Table 1:** Patients’ demographical data.

		Mean±SD	Min-Max
Age (years)		61.0±11.6	35-84
		n	%
Sex	Male	50	76.9
	Female	15	23.1
Profession	Housewife	15	23.1
	Retired	19	29.2
	Self-employed	17	26.2
	Others	14	21.5

SD=standard deviation

Statistical analysis was calculated with the Statistical Package for the Social Sciences (SPSS) software, version 15.0. Analysis of quantitative variables in dependent groups was calculated with the Friedman’s test. Subgroup analysis was done with Wilcoxon’s test. Student *t*-test and Mann-Whitney U test were used to compare the independent groups. The correlation between quantitative variables was analyzed with Spearman’s correlation analysis test. Statistical alpha significant value accepted was *P*<0.05.

## RESULTS

**Fig. 1:**
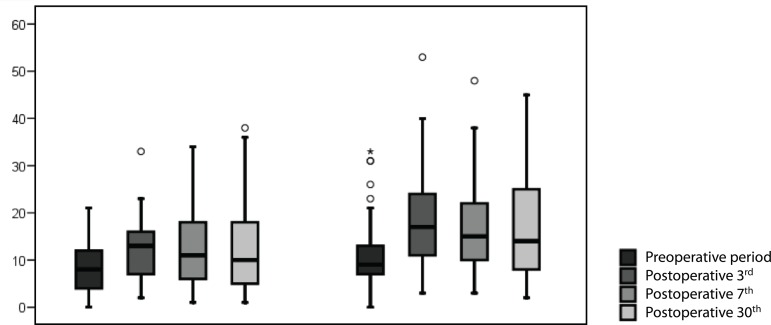
Statistical analysis of the Beck’s Depression Inventory and Beck’s Anxiety Inventory results.

Both BDI and BAI test results on preoperative period and on postoperative 3^rd^, 7^th^, and 30^th^ days are shown on Table 2. All the scores are analyzed statistically. Average BDI was 8.12±5.44 preoperatively, which increased to 12.43±6.36 on the postoperative 3^rd^ day. Average BAI was 11.28±7.28 preoperatively, which increased to 18.26±9.63 on the postoperative 3^rd^ day. We found that symptoms of depression and anxiety levels were higher in the postoperative period than in the preoperative period (*P*<0.001). Statistical analysis of both BDI and BAI tests is seen on Figure 1.

**Table 2:** Statistical analysis of the anxiety and depression levels. Beck’s depression and anxiety scores increased significantly in the postoperative period compared to the preoperative time.

		Mean±SD	Min-Max	Median
Beck’s Depression Inventory	Preoperative period	8.12±5.44	0-21	8
	Postoperative 3^rd^ day	12.43±6.36	2-33	13
	Postoperative 7^th^ day	11.66±6.95	1-34	11
	Postoperative 30^th^ day	12.29±9.08	1-38	10
	P*	<0.001		
Beck’s Anxiety Inventory	Preoperative period	11.28±7.28	0-33	9
	Postoperative 3^rd^ day	18.26±9.63	3-53	17
	Postoperative 7^th^ day	17.17±9.77	3-48	15
	Postoperative 30^th^ day	16.89±11.19	2-45	14
	P*	<0.001		

* Friedman’s analysis

SD=standard deviation

However after the postoperative 3^rd^ day, there was no statistical changing in depression and anxiety levels compared with postoperative 7^th^ and 30^th^ days. Average BDI were 11.66±6.95 and 12.29±9.08 on postoperative 7^th^ and 30^th^ days, respectively. Average BAI were 17.17±9.77 and 16.89±11.19 on postoperative 7^th^ and 30^th^ days, respectively. The results of this subgroup analysis are presented on Table 3.

**Table 3:** Subgroup analysis: both anxiety and depression levels increased on postoperative 3^rd^, 7^th^, and 30^th^ days compared with preoperative test results (*P*<0.001).

			P**
Beck’s Depression Inventory	Preoperative period	Postoperative 3^rd^ day	<0.001
		Postoperative 7^th^ day	<0.001
		Postoperative 30^th^ day	<0.001
	Postoperative 3^rd^ day	Postoperative 7^th^ day	0.011
		Postoperative 30^th^ day	0.511
	Postoperative 7^th^ day	Postoperative 30^th^ day	0.371
Beck’s Anxiety Inventory	Preoperative period	Postoperative 3^rd^ day	<0.001
		Postoperative 7^th^ day	<0.001
		Postoperative 30^th^ day	<0.001
	Postoperative 3^rd^ day	Postoperative 7^th^ day	0.037
		Postoperative 30^th^ day	0.058
	Postoperative 7^th^ day	Postoperative 30^th^ day	0.262

** Wilcoxon’s analysis with Bonferroni correction *P*<0.0083

We show the percentage of patients with symptoms of anxiety and depression in preoperative and postoperative periods. All the changes in the follow-up were statistically significant compared to the preoperative evaluation (BDI score on preoperative 1^st^ day: *P*=0.001; all other comparisons: *P*<0.001). The results of this analysis are presented on Table 4.

**Table 4:** Percentage of patients with symptoms of anxiety and depression in the preoperative and postoperative periods.

	Preoperative period		Postoperative 3^rd^ day		Postoperative 7^th^ day		Postoperative 30^th^ day	
	n	%	n	%	n	%	n	%
**BDI scores**								
<10 normal	40	61.5	23	35.4	26	40.0	31	47.7
10-16 mild depressive symptoms	20	30.8	26	40.0	20	30.8	17	26.2
17-29 moderate depressive symptoms	5	7.7	15	23.1	18	27.7	15	23.1
30-63 severe depressive symptoms	-	-	1	1.5	1	1.5	2	3.1
**BAI scores**								
<8 normal	21	32.3	4	6.2	9	13.8	12	18.5
8–15 mild anxiety symptoms	29	44.6	25	38.5	24	36.9	30	46.2
16–25 moderate anxiety symptoms	11	16.9	23	35.4	20	30.8	7	10.8
26–63 severe anxiety sympto	4	6.2	13	20.0	12	18.5	16	24.6

BAI=Beck’s Anxiety Inventory; BDI=Beck’s Depression Inventory

Statistical correlation of depression and anxiety in different ages (Table 5), genders (Table 6), and professions (Table 7) were also evaluated. But no significant correlation was found between these data separately and BDI and BAI (*P*>0.05).

**Table 5:** Statistical correlation between age and Beck’s depression and Beck’s anxiety test values (no significant value; *P*>0.05).

		Age	
		rho	P*
Beck’s Depression Inventory	Preoperative period	0.129	0.305
	Postoperative 3^rd^ day	0.185	0.139
	Postoperative 7^th^ day	0.151	0.230
	Postoperative 30^th^ day	0.157	0.212
Beck’s Anxiety Inventory	Preoperative period	0.103	0.413
	Postoperative 3^rd^ day	0.093	0.459
	Postoperative 7^th^ day	0.110	0.382
	Postoperative 30^th^ day	0.039	0.759

* Spearman’s correlation analysis

**Table 6:** Statistical correlation between gender and Beck’s depression and Beck’s anxiety test values (no significant value; *P*>0.05).

		Male		Female		P*
		Mean±SD	Median	Mean±SD	Median	
Beck’s Depression Inventory	Preoperative period	8.58±5.57	8.5	6.60±4.82	5	0.302
	Postoperative 3^rd^ day	12.64±6.07	13	11.73±7.41	11	0.632**
	Postoperative 7^th^ day	11.74±6.61	11	11.40±8.22	11	0.869**
	Postoperative 30^th^ day	12.24±8.91	10	12.47±9.95	9	0.913
Beck’s Anxiety Inventory	Preoperative period	11.66±7.80	9	10.00±5.22	9	0.870
	Postoperative 3^rd^ day	18.64±8.97	17.5	17.00±11.82	14	0.337
	Postoperative 7^th^ day	17.44±9.51	17	16.27±10.91	15	0.449
	Postoperative 30^th^ day	17.44±11.08	15	15.07±11.74	12	0.289

* Mann-Whitney U test;

** Student *t*-test

SD=standard deviation

**Table 7:** Statistical correlation between profession and Beck’s depression and Beck’s anxiety test values(no significant value; *P*>0.05).

	Housewife		Retired		Self-employed		Others		P*
	Mean±SD	Median	Mean±SD	Median	Mean±SD	Median	Mean±SD	Median	
**Beck’s Depression Inventory**									
Preoperative period	6.60±4.82	5	9.47±5.99	11	9.06±5.56	9	6.79±4.93	5	0.301**
Postoperative 3^rd^ day	11.73±7.41	11	13.74±6.57	15	12.59±5.08	13	11.21±6.60	9.5	0.930
Postoperative 7^th^ day	11.40±8.22	11	12.74±7.27	11	11.65±5.69	11	10.50±6.97	8	1.000
Postoperative 30^th^ day	12.47±9.95	9	14.32±10.42	13	12.00±7.66	10	9.71±7.95	6.5	0.406
**Beck’s Anxiety Inventory**									
Preoperative period	10.00±5.22	9	11.74±8.74	10	12.35±7.87	9	10.71±6.74	8.5	0.965
Postoperative 3^rd^ day	17.00±11.82	14	19.32±9.64	18	17.12±6.05	17	19.57±11.18	17	0.525
Postoperative 7^th^ day	16.27±10.91	15	18.11±9.13	18	16.71±7.30	17	17.43±12.57	12.5	0.965
Postoperative 30^th^ day	15.07±11.74	12	17.53±10.86	15	17.71±9.75	15	17.00±13.51	9	0.948

* Kruskal-Wallis test;

** One-way ANOVA

ANOVA=analysis of variance; SD=standard deviation

## DISCUSSION

In this study, we aimed to evaluate unipolar symptoms of depression and anxiety levels in CABG patients. Unipolar depression is defined as depressed mood and/or loss of interest or pleasure. Gehi et al. reported that 15% to 20% prevalence of unipolar depression among CABG surgery patients is consistent with that found generally among cardiac patients^[4]^. In our study, unipolar symptoms of depression and anxiety levels in CABG patients were at the same rate.

Studies indicate that the number of CABG surgery patients affected by any depression (*i.e.*, major, minor, or dysthymia) is approximately between 30% and 40%^[5]^. However, some patients may develop new depressive symptoms over the course of recovery from surgery, in the postoperative period. McKhann et al.^[6]^ showed that 13% and 9% of 124 CABG patients at one-month and twelve-month follow-up, respectively, reported clinical relevant depressive symptoms, not evident at the time of surgery. In our study, depression in the postoperative period was significantly higher than in the preoperative period. We think that the higher rates of the depression on the postoperative period detected in our study might be associated with new depressive symptoms. Interestingly, the high prevalence of depression in CAD patients is not explained by cardiac disease severity or CAD-related functional impairments^[7]^.

Anxiety is a general term for several disorders that cause nervousness, fear, apprehension, and worrying. Some anxiety helps us react to stresses or potential threats. The most common anxiety symptoms are hot and cold flushes, shaking, and tachycardia^[8]^.

Anxiety increases before the CABG surgery and is particularly high while on the waiting list with an unknown surgery date^[8]^. The indication for CABG is particularly disturbing, since the heart is culturally regarded as the central organ of the body, the source of life and of the emotions^[9]^. As the time for surgery draws closer, the patients’ emotional reactions intensify, as shown in their behavior, symptoms, and, when given the opportunity, in words. The majority of patients with an indication for CABG report that fear, anxiety, and uncertainty with respect to the future are more distressing than the chest pain of the cardiac disease^[10]^. Many studies have shown that depression and anxiety have been more identified in the preoperative period than in the postoperative period because the preoperative duration was long and uncertain^[11]^. According to a study: preoperative evaluation comprehends the time when patients come to the hospital for a preoperative clinical examination, this is an average of 29 days. In this study, it was accepted an average of seven days in the postoperative period^[3]^. In our study, depression and anxiety levels in the postoperative period were significantly higher than in the preoperative period. We think that the higher rates of depression in the postoperative period detected in our study might be associated with the fact that the preoperative period of our patients is short and their postoperative period is long; the mean preoperative period of our patients is two days and their postoperative period is five days. In a study, the mean length of hospital stay for 19,522 CABG patients was 12.48 days (standard deviation = 10.94)^[12]^. In our study, the mean hospital stay was 7,15 days.

Some authors have recommended depression and anxiety screening following CABG surgery as a way to improve pathways to recovery^[13]^. The follow-up period was 30 days in our study.

Many studies have shown that early psychological management may be associated with a reduction of length of hospital stay, analgesic use, and post-surgical morbidity^[14]^, and may also help patients adopt more effective coping strategies in their everyday lives^[15]^.

Kazukauskiene et al. found out that mental distress factors and symptoms of depression are strongly associated with exercise capacity, both at the beginning and after exercise-based cardiac rehabilitation in patients with CAD^[16]^. In this study, we did not provide early psychological management to patients. However, we think that length of hospital stay, analgesic use, and post-surgical morbidity will decrease when mental support is provided.

Wellenius et al. suggest that in patients who had undergone previous CABG surgery, depressive symptoms were associated with higher risk of atherosclerotic progression in saphenous vein grafts. Their analysis provides prospective evidence for a direct association between depressive symptoms and atherosclerotic progression^[17]^.

Frasure-Smith et al.^[18]^ determined one-year survival status for myocardial infarction in 887 patients. They indicate that the relationship between depression and cardiac mortality decreased with increasing support.

Other psychological factors besides depression and anxiety have been reported to predict surgical outcomes. For example, optimism has been reported to correlate with a lower readmission rate six months after CABG, independently from sociodemographic and medical variables^[19]^. Conversely, pessimistic tendencies predicted greater psychological distress (anxiety, depression), greater functional restriction, and ineffective coping strategies during a 20-month postoperative follow-up period^[20]^. Everson et al. indicated that high hopelessness predicted incident myocardial infarction, and moderate hopelessness was associated with incident cancer^[21]^. Our study is not including primary psychological therapy, psychological drugs users, or psychological diagnosis.

BDI and BAI tests are widely used in cardiac samples. The measurements in these tests consist of two main factors: somatic and cognitive symptoms^[22]^. Many of the symptoms that BDI considers as depression are characteristic of patients with heart disease. One of five patients with heart disease has symptoms that BDI considers as depression^[23]^. It is known that the patients selected in our study did not have any psychological illness and did not use psychological drugs before the operation. The symptoms of depression in our patients were more frequent after the surgery, therefore, we did not think that these symptoms were due to heart disease.

## CONCLUSION

Both depression and anxiety appear to cause morbidity risks, although their behavioral and biological mechanisms are poorly understood. In all CABG surgery patients, depression and anxiety levels increase during the postoperative period. Careful routine evaluation of these psychological symptoms must be carried out and the symptoms detected and treated as part of the preoperative workup, similarly in importance to smoking and hypertension.

Statistical correlation of depression and anxiety in different ages, genders, and professions were evaluated too. But we did not found a significant correlation between them.

**Table:** 

Author's roles & responsibilities	
META	Substantial contributions to the conception or design of the work; or the acquisition, analysis, or interpretation of data for the work; final approval of the version to be published
